# Recent advances in neuromuscular block during anesthesia

**DOI:** 10.12688/f1000research.13169.1

**Published:** 2018-02-09

**Authors:** Martijn Boon, Christian Martini, Albert Dahan

**Affiliations:** 1 Department of Anesthesiology , Leiden University Medical Center, Leiden, Netherlands

**Keywords:** deep neuromuscular block, sugammadex, postoperative residual curarisation, surgical rating scale

## Abstract

Muscle relaxation is a routine part of anesthesia and has important advantages. However, the lingering effects of muscle relaxants in the postoperative period have historically been associated with postoperative adverse events. Neuromuscular reversal, together with neuromuscular monitoring, is a recognized strategy to reduce the rate of postoperative residual relaxation but has only marginally improved outcome in the past few decades.

Sugammadex, a novel reversal agent with unique encapsulating properties, has changed the landscape of neuromuscular reversal and opened up new opportunities to improve patient care. By quickly and completely reversing any depth of neuromuscular block, it may reduce the rate of residual relaxation and improve respiratory recovery. In addition, sugammadex has made the use of deep neuromuscular block possible during surgery. Deep neuromuscular block may improve surgical working conditions and allow for a reduction in insufflation pressures during selected laparoscopic procedures. However, whether and how this may impact outcomes is not well established.

## Introduction

**Figure 1.**
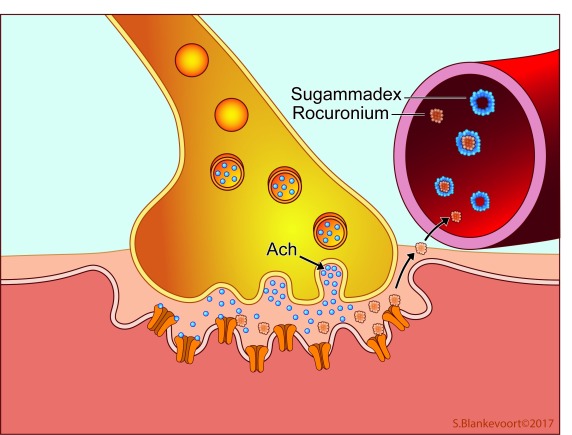
Neuromuscular transmission and blockage at the neuromuscular junction. Ach, acetylcholine.

Muscle relaxants or neuromuscular blocking agents (NMBAs), introduced in 1942 by Griffith and Johnson, revolutionized the practice of anesthesiology ^1^. NMBAs block neuromuscular transmission at the neuromuscular junction by binding to the postsynaptic nicotinergic acetylcholine receptor. This renders these receptors unavailable for acetylcholine-mediated neuromuscular signal transmission (see Figure 1). In practice, NMBAs enable anesthesiologists to temporarily paralyze patients during anesthesia. The introduction of NMBAs in anesthesia meant that optimal surgical conditions (i.e. by ensuring an immobile patient) could be achieved with lower doses of volatile or intravenous anesthetics, improving hemodynamic stability. Consequently, the induction of muscle relaxation became an established part of the classic anesthesia triad, alongside unconsciousness (hypnosis) and pain relief ^2^. However, like most medication, NMBAs are not devoid of disadvantages. Lingering effects of NMBAs in the postoperative period, also known as postoperative residual curarization (PORC), may cause life-threatening respiratory complications in the first few hours after surgery ^3^. In 1954, Beecher *et al*. were the first to note a sixfold increase in anesthesia-related mortality when NMBAs were used ^4^. Despite the development of shorter-acting agents and neuromuscular monitoring techniques, NMBAs continue to be associated with severe adverse events after anesthesia, even today ^5, 6^.

## Reversal of neuromuscular block

Currently, two concepts of neuromuscular reversal exist. A moderate neuromuscular block (NMB) (see below) is traditionally reversed with an acetylcholinesterase inhibitor such as neostigmine. These drugs increase the amount of acetylcholine in the neuromuscular junction by inhibiting the enzyme acetylcholinesterase. The increased levels of acetylcholine compete with the NMBA molecules for the postsynaptic nicotine receptors (i.e. competitive antagonism) and tip the balance towards enhanced signal transmission. Encapsulation of NMBA molecules by sugammadex represents a novel reversal strategy. Sugammadex is a modified γ-cyclodextrin, which is able to selectively bind free plasma NMBA molecules ( Figure 1) ^7^. Encapsulation by sugammadex immediately inactivates these NMBA molecules, rendering them permanently unavailable for redistribution to the neuromuscular junction ^8^. Sugammadex produces rapid and safe reversal of the commonly used non-depolarizing NMBAs rocuronium and vecuronium ^9, 10^. It encapsulates and consequently inactivates these NMBA molecules on a one-to-one basis and is able to reverse both moderate and deep or even intense levels of NMB (see below) ^11– 13^. Importantly, sugammadex reversal is much faster and more intense than reversal with acetylcholinesterase inhibitors ^14^. For example, the average time for reversal of a moderate neuromuscular block is 2.7 minutes after administration of 2 mg.kg ^-1^ sugammadex compared to 17.9 minutes after administration of 50 μg.kg ^-1^ neostigmine ^15^. In addition, sugammadex is well tolerated by patients and is devoid of cholinergic side effects ^14, 16^. Sugammadex has been available in Europe since 2008 and was approved by the FDA for use in the USA in 2015.

Although the introduction of sugammadex represents a great improvement in the reversal of NMB, there are some important aspects that deserve consideration. First, only NMB induced by rocuronium, vecuronium, and pancuronium can be reversed with sugammadex, leaving acetylcholinesterase inhibitors the only choice for reversal of the other NMBAs, such as cisatracurium. In the future, new broad-spectrum encapsulating agents may become available for all NMBAs ^17^. Second, the cost of sugammadex is significant (in the Netherlands, one ampoule of 200 mg costs 78 euro). It is unclear whether sugammadex reversal leads to an improved postoperative outcome that justifies its cost. The same holds true for another emerging area of interest made possible by sugammadex, which is the application of a deep NMB during anesthesia. With the introduction of sugammadex, the use of a deep NMB during surgery is now possible without the fear of prolonged recovery times. Deep NMB may improve surgical working conditions for some procedures and allows for a reduction in insufflation pressures during laparoscopic surgeries ^18– 21^. However, the impact of deep NMB on patient outcome is still unclear.

## Monitoring depth of neuromuscular block

**Figure 2.**
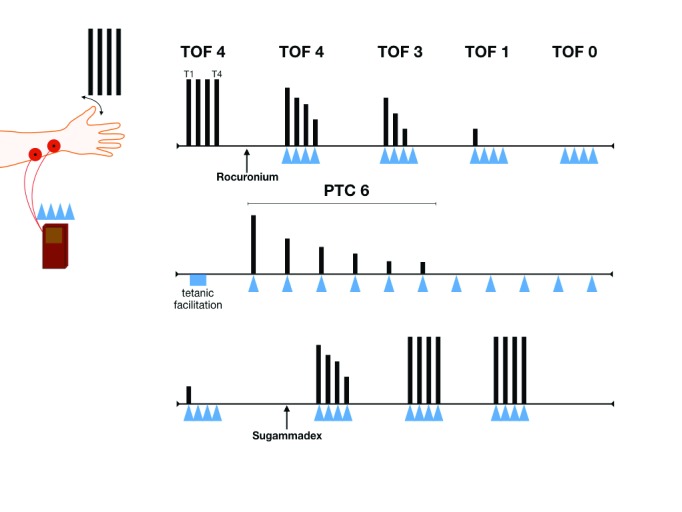
Neuromuscular monitoring. PTC, post tetanic count; TOF, train of four.

Neuromuscular monitoring during anesthesia is most commonly performed using the train of four (TOF) method ^22^. TOF peripheral nerve monitors (such as the TOF-Watch ^TM^ monitor) are usually applied at the distal forearm to stimulate the ulnar nerve. Here, four consecutive supramaximal electrical stimuli (a TOF) will evoke contractions (twitches) at the musculus adductor pollices of the thumb. Under normal conditions, the amplitude of all four motor responses will be equal. With an increasing degree of NMB (induced by non-depolarizing NMBAs), the amplitude of the latter twitches decreases, relative to the first twitches, a phenomenon called fade. Eventually, as NMB increases, all twitches will become absent (see Figure 2). Thus, the number of detectable thumb twitches and the degree of fading correspond with the intensity of the NMB. The degree of fading can be further expressed as a ratio, by dividing the motor response of the fourth twitch (T4) to the first twitch (T1), i.e. the T4:T1 ratio or the so-called TOF ratio. Available evidence indicates that the NMB has to be recovered to a TOF ratio of 0.9 or greater to allow for safe extubation of the patient ^23– 27^.

When high doses of NMBAs are given, measurement of the NMB at the ulnar nerve will show zero thumb twitches (TOF equals zero). To measure the degree of NMB in this instance, a tetanic stimulus of 50 Hz for five seconds is applied to the ulnar nerve. The tetanic stimulus causes a large amount of acetylcholine to be released in the neuromuscular junction. This tetanic facilitation is subsequently followed by 15 single electrical stimuli delivered at one-second intervals. The number of measured thumb twitches make up the post tetanic count ** (PTC) ^28^. For example, when six thumb twitches are observed following the tetanic facilitation, the PTC equals six (see Figure 2). With TOF and PTC measurements, the depth of the NMB can be classified as follows ^29^: (1) moderate NMB: TOF one to three out of four twitches; (2) deep NMB: TOF zero twitches and PTC more than zero twitches; (3) intense NMB: TOF zero and PTC zero twitches. Note that, in practice, an intense NMB is present only at the beginning of anesthesia following the induction dose of NMBA. Thereafter, NMB is allowed to recover to a deep or moderate NMB, which can be maintained to preserve adequate surgical working conditions, depending on the type of surgery.

## Postoperative residual curarization

Full recovery of NMB at the end of anesthesia is essential for the return of adequate respiration and upper airway muscle function ^3, 30, 31^. By definition, PORC is present when some level of NMB (TOF ratio <0.9) persists after extubation. This can readily occur, as most NMBAs have much longer recovery times than the often short-acting opioids and hypnotics used during general anesthesia. In addition, it is impossible to predict recovery of NMB with pharmacologic (PKPD) reasoning, as recovery times of NMBAs display a wide inter-individual variation ^32, 33^.

Residual curarization negatively affects pulmonary and upper airway muscle function. It promotes upper airway collapse and ventilatory compromise. This is relevant, as even a small degree of residual curarization (e.g. TOF ratio between 0.6 and 0.9) is associated with increased upper airway collapsibility and dysfunction of pharyngeal and upper esophageal sphincter muscles ^23, 27^. Additionally, NMBAs directly attenuate the hypoxic ventilatory response due to blocking of nicotinergic acetylcholine receptors in the carotid bodies ^24^. Inhibition of the hypoxic ventilatory response renders patients at increased risk for hypoxia. Owing to these effects, PORC is highly associated with postoperative respiratory complications ^3, 30^. Unfortunately, incidences of PORC are substantial and range between 20 and 60% of patients in the post-anesthesia care unit (PACU) ^31, 34, 35^. Use of a neuromuscular monitor and adequate reversal of NMB are essential strategies that will reduce the incidence of PORC.

## Prevention of postoperative residual curarization

With the use of neostigmine and other acetylcholinesterase inhibitors, a variable degree of residual NMB often persists ^36^. It is therefore not surprising that the effect of NMB reversal with neostigmine on postoperative respiratory complications and outcome is, at best, ambiguous. Increasing evidence shows that NMB reversal with neostigmine (without the guidance of a TOF watch) does not improve postoperative respiratory safety ^37^ and may even be associated with increased rates of atelectasis ^38^, hypoxemia ^39^, and, consequently, reintubation ^40^. There are several explanations for these findings. Timely administration and exclusive reversal of a moderate NMB are important for successful reversal. Evidently, this requires adequate neuromuscular monitoring. In addition, time to full reversal following neostigmine treatment displays wide between-patient variations and is unpredictable. Sugammadex has the potential to do better in both respects, as it allows for fast, complete, and predictable reversal of both moderate and deep NMB ^15, 16, 41, 42^. Emerging evidence shows that NMB reversal with sugammadex reduces the rate of postoperative residual curarization compared to reversal with neostigmine (see Table 1) ^36, 39, 43^. A recent investigation reported a 0% PORC rate in patients reversed with sugammadex versus 46% in those who received neostigmine ^43^. These results are promising; however, in an unmonitored setting, PORC after sugammadex reversal still occurred in 4% of patients ^36, 39, 44^. This highlights the need for adequate neuromuscular monitoring in any setting where NMBAs are used, regardless of the type of reversal agent.

**Table 1.** Studies comparing sugammadex and neostigmine on incidence of postoperative residual curarization and pulmonary outcome.

Author	Year	Design	Comparison	Monitoring	PORC	Pulmonary outcome
Kotake ^44^	2013	Prospective observational	Sugammadex versus neostigmine	No	4.3% versus 23.9% **	UA
Ledowski ^47^	2014	Retrospective cohort	Sugammadex versus neostigmine	Available	UA	Reduced pulmonary outcome score in ASA 3–4 patients **
Brueckmann ^43^	2015	RCT	Sugammadex versus neostigmine	Available	0% versus 43.3% **	Respiratory disorders: 1.4% versus 6.5% #
						Hypoxemia: 1.4% versus 2.6% #
Boon ^39^	2016	RCT	Sugammadex versus neostigmine	No	4% versus 70% **	Lowest O _2_ saturation: 93.3 versus 96.8% **
Nemes ^36^	2017	RCT	Sugammadex versus neostigmine	No	3.7% versus 15.4% #	UA

ASA, American Society of Anesthesiologists; PORC, postoperative residual curarization (train of four [TOF] ratio <0.9 after extubation); RCT, randomized controlled trial; UA, unavailable. * *p*<0.05 ** *p*<0.001 # *p*>0.05

We argue that NMB reversal with sugammadex will decrease the incidence of postoperative pulmonary complications by causing complete recovery of ventilatory muscle strength. This was shown in two studies in healthy volunteers. Sugammadex reversal led to a higher degree of diaphragmatic and intercostal muscle activation and higher arterial pO _2_ values compared to neostigmine reversal ^45, 46^. In addition, it is likely that sugammadex will allow for a better return of the hypoxic ventilatory drive, which is attenuated at very low levels of residual neuromuscular block ^24^. Especially in vulnerable patients, such as the obese and elderly, full recovery of the ventilatory muscles and hypoxic ventilatory reflex is crucial to prevent pulmonary complications. Initial evidence from a retrospective study shows that sugammadex reversal was associated with reduced incidence of pulmonary complications in elderly ASA three and four patients compared to reversal with neostigmine ^47^. In a small prospective study, sugammadex reversal was associated with fewer hypoxemic events in the PACU compared to neostigmine reversal ^39^. The current evidence is far from complete, and future prospective studies should determine the exact value of sugammadex in improving post-anesthesia pulmonary outcome.

## Deep neuromuscular block: prevention of diaphragmatic contractions and optimized surgical conditions

The most important advantages of a deep NMB over a moderate block are the full relaxation of the abdominal wall musculature and diaphragm. This results in a significant improvement in surgical conditions, especially of procedures confined to a narrow space, such as laparoscopic surgery. Both abdominal wall muscles and the diaphragm are more resistant to NMBAs compared to the reference muscle musculus adductor pollicis ^48– 50^. A deep NMB is required to fully relax these muscle groups. For example, Fernando and colleagues showed that a deep NMB is required to silence the diaphragm in response to stimulation of the carina ^48^. Similarly, Werba and colleagues showed that diaphragmatic responses evoked by tracheal suctioning led to coughing, bucking, and elevated intracranial pressures in neurosurgical patients, unless deep NMB was applied ^49^. In addition, during laparoscopic surgery, efferent activation of the diaphragm from brainstem chemosensitive respiratory centers may occur as a result of elevated arterial pCO _2_ levels (due to CO _2_ insufflation). Only in deep NMB are these diaphragmatic contractions effectively prevented.

Martini *et al*. assessed the effect of deep versus moderate NMB on surgical conditions during laparoscopic retroperitoneal urologic surgery ^19^. They developed the validated five-point Leiden surgical rating scale (L-SRS, 0–5; extremely poor to optimal working conditions) to quantify the quality of the surgical field as experienced by the surgeon at various points during the procedure ^19, 20, 51^. The study showed an improvement of 0.7 L-SRS points (mean L-SRS 4.0 versus 4.7) when deep NMB was applied, an improvement deemed clinically significant by the surgical team ^19^. In many other procedures, a similar effect of deep NMB was found ^18, 20, 21, 52– 54^, but it is important to acknowledge that some studies found no effect of deep NMB on surgical conditions (see Table 2) ^55^. A recent meta-analysis confirmed the positive effect of a deep NMB on surgical conditions and reduced postoperative pain scores; however, significant heterogeneity between the included studies reduces the overall quality of evidence ^56^. It is important to realize that other factors such as deep anesthesia may positively affect surgical working conditions. However, deep anesthesia, although applicable, is associated with less hemodynamic stability and prolonged recovery times.

**Table 2.** Studies assessing deep NMB on surgical conditions during open and laparoscopic surgery (normal pressure pneumoperitoneum).

Author	Specialty	Control	Intervention	Scale	Mean score	% unacceptable surgical conditions
Martini ^19^	Urology (laparoscopy)	Moderate NMB	Deep NMB	L-SRS	4.0 versus 4.7 **	18% versus 1%
Yoo ^21^	Urology (laparoscopy)	Moderate NMB	Deep NMB	L-SRS	3.0 versus 4.0 **	UA
Boon ^52^	Urology (laparoscopy)	Deep NMB + hypercapnia	Deep NMB + hypocapnia	L-SRS	4.84 versus 4.77 #	1 versus 1%
Torensma ^20^	Bariatric surgery (laparoscopy)	Moderate NMB	Deep NMB	L-SRS	4.2 versus 4.8 **	UA
Baete ^55^	Bariatric surgery (laparoscopy)	Moderate NMB	Deep NMB	L-SRS	4.1 versus 3.9 #	UA
Madsen ^62^	Gynecology (laparoscopy)	No NMB	Deep NMB	1 (optimal) – 4 (unacceptable)	1.7 versus 1.0 *	UA
Blobner ^18^	General surgery (laparoscopy)	No NMB	Deep NMB	0 (not acceptable) – 100 (excellent)	UA	0 versus 28% *
Rosenberg ^54^	General surgery (laparoscopy)	Moderate NMB	Deep NMB	0 (poor) – 10 (excellent)	6.8 versus 7.9 *	UA
Madsen ^53^	General surgery (laparotomy)	Moderate NMB	Deep NMB	L-SRS	4.0 versus 4.75 **	17 versus 49% **

L-SRS: Leiden surgical rating scale (1: extremely poor – 5: optimal) ^19^; NMB, neuromuscular block; UA, unavailable. * *p*<0.05 ** *p*<0.001 # *p*>0.05

Adversaries of deep NMB claim that the gains in surgical conditions with deep NMB are modest at best and are not worth the extra effort and cost of the reversal agents (sugammadex) ^57, 58^. We argue that the observed differences in L-SRS are clinically relevant, the incidence of suboptimal conditions is greatly reduced during deep NMB (especially the occurrence of sudden diaphragmatic contractions) ^18– 20, 52^, and, most important, deep NMB is associated with less postoperative pain and a lesser incidence of unplanned 30-day readmission ^20, 59^.

Finally, there are indications that a deep NMB allows for lower intra-abdominal pressure during laparoscopic surgery. Reduced insufflation pressure is associated with less postoperative pain ^60^. Deep NMB might cause an increase in abdominal wall compliance and consequently an increase in intra-abdominal space ^61, 62^. However, while various studies indeed show that deep NMB allows titration to lower intra-abdominal pressures with still-acceptable surgical conditions, the gain in intra-abdominal space may be marginal ^62^, and the incidence of unacceptable surgical conditions remained substantially higher than under standard pressures. Hence, the feasibility of low-pressure pneumoperitoneum needs further investigation.

## Conclusions

NMBAs certainly have important advantages but also serious disadvantages. Postoperative residual curarization is an important threat, especially in patients who are not adequately reversed or monitored. An important new development is the introduction of the reversal agent sugammadex. Sugammadex may help reduce the incidence of postoperative residual curarization and improve postoperative respiratory recovery. In addition, sugammadex enables the use of a deep NMB during general anesthesia. While the deep NMB has been shown to improve surgical conditions and reduce postoperative pain in a variety of procedures, its place in anesthesia is not yet fully determined.
